# Bihemispheric-tDCS and Upper Limb Rehabilitation Improves Retention of Motor Function in Chronic Stroke: A Pilot Study

**DOI:** 10.3389/fnhum.2016.00258

**Published:** 2016-06-09

**Authors:** Alicia M. Goodwill, Wei-Peng Teo, Prue Morgan, Robin M. Daly, Dawson J. Kidgell

**Affiliations:** ^1^Institute for Physical Activity and Nutrition, Deakin UniversityMelbourne, VIC, Australia; ^2^Department of Physiotherapy, Faculty of Medicine, Nursing and Health Science, Monash UniversityFrankston, VIC, Australia; ^3^Department of Rehabilitation, Nutrition and Sport, School of Allied Health, La Trobe UniversityMelbourne, VIC, Australia

**Keywords:** bihemispheric-tDCS, chronic stroke, corticospinal excitability, intracortical inhibition, motor function, rehabilitation

## Abstract

**Background**: Single sessions of bihemispheric transcranial direct-current stimulation (bihemispheric-tDCS) with concurrent rehabilitation improves motor function in stroke survivors, which outlasts the stimulation period. However few studies have investigated the behavioral and neurophysiological adaptations following a multi-session intervention of bihemispheric-tDCS concurrent with rehabilitation.

**Objective**: This pilot study explored the immediate and lasting effects of 3-weeks of bihemispheric-tDCS and upper limb (UL) rehabilitation on motor function and corticospinal plasticity in chronic stroke survivors.

**Methods**: Fifteen chronic stroke survivors underwent 3-weeks of UL rehabilitation with sham or real bihemispheric-tDCS. UL motor function was assessed via the Motor Assessment Scale (MAS), Tardieu Scale and grip strength. Corticospinal plasticity was indexed by motor evoked potentials (MEPs), cortical silent period (CSP) and short-interval intracortical inhibition (SICI) recorded from the paretic and non-paretic ULs, using transcranial magnetic stimulation (TMS). Measures were taken at baseline, 48 h post and 3-weeks following (follow-up) the intervention.

**Results**: MAS improved following both real-tDCS (62%) and sham-tDCS (43%, *P* < 0.001), however at 3-weeks follow-up, the real-tDCS condition retained these newly regained motor skills to a greater degree than sham-tDCS (real-tDCS 64%, sham-tDCS 21%, *P* = 0.002). MEP amplitudes from the paretic UL increased for real-tDCS (46%: *P* < 0.001) and were maintained at 3-weeks follow-up (38%: *P* = 0.03), whereas no changes were observed with sham-tDCS. No changes in MEPs from the non-paretic nor SICI from the paretic UL were observed for either group. SICI from the non-paretic UL was greater at follow-up, for real-tDCS (27%: *P* = 0.04). CSP from the non-paretic UL increased by 33% following the intervention for real-tDCS compared with sham-tDCS (*P* = 0.04), which was maintained at 3-weeks follow-up (24%: *P* = 0.04).

**Conclusion**: bihemispheric-tDCS improved retention of gains in motor function, which appears to be modulated through intracortical inhibitory pathways in the contralesional primary motor cortex (M1). The findings provide preliminary evidence for the benefits of bihemispheric-tDCS during rehabilitation. Larger clinical trials are warranted to examine long term benefits of bihemispheric-tDCS in a stroke affected population.

## Introduction

Transcranial direct-current stimulation (tDCS) is a non-invasive technique that modulates neuronal excitability in a polarity-specific manner (Nitsche et al., 2008). Anodal-tDCS (a-tDCS) of the primary motor cortex (M1) increases corticospinal excitability, whilst cathodal-tDCS (c-tDCS) exerts an inhibitory effect (Nitsche et al., 2005, 2008; Bastani and Jaberzadeh, 2012). Additionally, bihemispheric-tDCS which involves placing the anode and cathode over both M1s simultaneously, has been shown to increase excitability of one hemisphere whilst suppressing the other (Mordillo-Mateos et al., 2012).

Recovery of upper limb (UL) function following a stroke is multifaceted and influenced by a combination of factors, including the extent of damage within the corticospinal pathway and abnormal interactions between the ipsi- and contralesional M1s (Boroojerdi et al., 1996; Stinear et al., 2007; Di Pino et al., 2014). In chronic stroke, maladaptive changes in ipsi- and contralesional M1 γ-aminobutyric acid (GABA)-mediated inhibition has been proposed as one potential model that may hinder functional recovery (Nowak et al., 2009). In animal models, disinhibition of the contralesional M1 is associated with impairments in both N-methyl-D-aspartate (NMDA) receptor binding along with GABA-mediated inhibition (Que et al., 1999; Reinecke et al., 1999). Specifically, disinhibition of the contralesional M1 may result in increased inhibition of the ipsilesional M1, impeding recovery of the paretic limb (Liepert et al., 2000b; Murase et al., 2004). Therefore, the application of bihemispheric-tDCS may serve to normalize excitatory and inhibitory corticospinal networks within both M1s, which may lead to lasting functional improvement in the paretic limb (Nowak et al., 2009; Feng et al., 2013).

In chronic stroke, preliminary evidence has demonstrated a preferential improvement in UL motor function following single sessions of bihemispheric-tDCS combined with physical therapy (Lefebvre et al., 2012, 2014), however there are currently limited studies investigating the lasting effects of multi-session interventions. It is well documented that repetition of motor training is important for the induction of corticospinal plasticity (Butefisch et al., 2000; Hayashi et al., 2005), and reflect mechanisms analogous to motor learning, such as long term potentiation (LTP; Butefisch et al., 2000). Moreover, LTP-like mechanisms have also been observed in healthy individuals following repetitive sessions of a-tDCS (Monte-Silva et al., 2013). Given that recovery following stroke is regarded as a form of motor learning (Krakauer, 2006), it is likely that repeated sessions of concurrent bihemispheric-tDCS and rehabilitation would be favorable for inducing corticospinal plasticity in this population. In chronic stroke patients, only 2 studies have prescribed 5 and 10 sessions of bihemispheric-tDCS combined with UL rehabilitation, reporting sustained improvements in motor function for up to 1 month (Lindenberg et al., 2010; Bolognini et al., 2011). Although both studies demonstrated improvements in activation of the ipsilesional M1 following bihemispheric-tDCS, transcranial magnetic stimulation (TMS) measures of corticospinal excitability and intracortical inhibition underscoring the lasting improvements in motor function were not assessed. In acute stroke patients, Di Lazzaro et al. (2014) performed a single session of bihemispheric-tDCS and constraint induced movement therapy (CIMT), reporting an enduring reduction in interhemispheric inhibition (IHI) for up to 3 months. Surprisingly, these neurophysiological adaptations did not correlate with any clinical improvement, which may be due to a ceiling affect for motor recovery in the acute stage of stroke. Based on the current literature, the neurophysiological mechanisms involved in the lasting clinical improvement observed following bihemispheric-tDCS and rehabilitation, remain inconclusive.

Therefore, the aim of this study was to quantify the immediate and lasting effects of a 3-week concurrent bihemispheric-tDCS and UL rehabilitation intervention on motor function in chronic stroke survivors. The secondary aim was to explore the adaptations in corticospinal excitability and inhibition within the ipsi- and contralesional M1. We hypothesized that concurrent bihemispheric-tDCS and UL rehabilitation would elicit greater improvements in motor function, which would accompany increased corticospinal excitability and inhibition in the ipsi- and contralesional M1 respectively. We further hypothesized that the addition of bihemispheric-tDCS would elicit longer-lasting improvements in UL function compared with UL rehabilitation alone.

## Materials and Methods

### Participants

**Figure 1:**
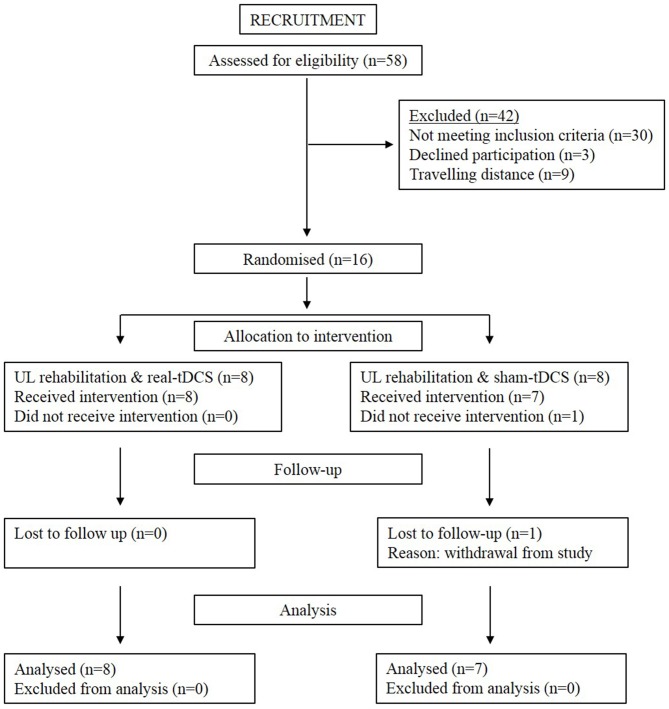
**Consort diagram depicting flow through the study from recruitment to analysis**.

Sixteen participants aged 18–90 years with a single, unilateral hemispheric ischemic or hemorrhagic stroke (>6-months clinically diagnosed and confirmed by imaging) were recruited into the study (Figure 1). Information noting the side of hemiparesis, stroke subtype and year of stroke was obtained through a screening questionnaire. Participants were excluded on the following: (1) a score of <2 or >15 out of 18 on the combined UL items of the Motor Assessment Scale (MAS); (2) pre-stroke UL disability; (3) other known neurological disorder; (4) excessive UL pain (including glenohumeral joint subluxation); (5) botulinum Toxin (BOTOX) injections <6-months; (6) medications known to directly influence corticospinal excitability; (7) severe mental health condition or cognitive impairment [Mini Mental State Examination (MMSE)] score <18]; and (8) contraindications to TMS/tDCS. The study was approved by the Deakin University Human Research Ethics Committee (2012-081), and all procedures were conducted in accordance to the Declaration of Helsinki.

### Experimental Design and Study Flow

This was a double-blinded randomized controlled trial consisting of a 3-week intervention and follow-up at week 6 (3-weeks post-intervention). Baseline measures of corticospinal excitability, grip strength, spasticity, and motor function were assessed. Thereafter, participants were systematically matched for MAS scores and then randomly allocated to real-tDCS or sham-tDCS with UL rehabilitation. Randomization was performed by a researcher independent to the study, using a computer-generated random numbers table in Excel. Both participants and the researcher were blinded to the group allocation. All participants then undertook 9 (3 sessions/week), 40 min, individually supervised UL training sessions, with real or sham bihemispheric-tDCS applied during the initial 20 min. The rehabilitation was designed in conjunction with an experienced neuro-physiotherapist and delivered by a trained exercise scientist, whom were both blinded from the intervention condition. Post and follow-up assessments of motor function, spasticity and corticospinal excitability and inhibition were administered 48 h and 3-weeks following the final training session.

### Assessment of Motor Function

#### MAS

UL motor function was assessed using the modified version of the MAS (Carr and Shepherd, 1989), which comprised of 18 tasks, split into 3 items corresponding to “arm”, “hand” and “advanced hand” activities. Each sub-section was scored out of 6 and summed to provide a total score out of 18.

#### Grip Strength

A maximal isometric contraction (MVIC) using a pre-calibrated strain gauge isometric dynamometer with a linear response in the 0–800 N range (ADInstruments, Bella Vista, Sydney, NSW, Australia) was used to quantify grip strength (N). Participants were seated in an armchair with their elbow flexed at 90° and forearm supported in pronation. With the wrist in an anatomically neutral position, participants were asked to squeeze the transducer maximally for 3 s, while maximal root mean square electromyography (rmsEMG) was recorded for 100 ms epoch during the asymptote of the MVIC. The highest of 3 MVIC trials was recorded.

#### Tardieu Scale

The Modified Tardieu Scale (Boyd and Graham, 1999) assessed spasticity in the distal UL. The researcher guided the patients arm through passive wrist and elbow extension as quickly as possible through the full available joint range. The quality of the muscle reaction was recorded from a 0–5 scale.

### UL Rehabilitation Intervention

**Figure 2:**
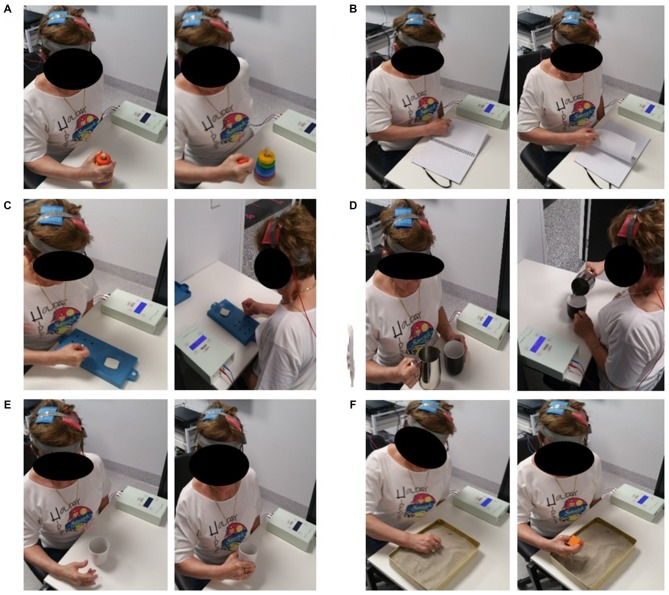
**One participant undertaking a selection of the above upper limb (UL) rehabilitation exercises during the application of bihemispheric transcranial direct-current stimulation (bihemispheric-tDCS).** Exercises shown include: grasping task using stacking blocks **(A)**; turning pages of a scrapbook **(B)**; nine-hole pegboard **(C)**; grasping saucepan and pouring water into a cup **(D)**; opening hand using finger and wrist extension to grasp and pick up cup **(E)**; feeling for blocks in sand and picking them up **(F)**. The picture on the left depicts the beginning of the task whilst the right shows the end range of the task.

Following a 5 min warm-up consisting of active UL range of motion, participants completed 4, individually tailored exercises for a total duration of approximately 6 min each. This consisted of 3 sets for each exercise and as many controlled repetitions as possible within 2 min, with a 30 s rest between sets and approximately 2–3 min between exercises. All exercises were standardized to target the distal UL musculature and training for participants was matched for volume, intensity and rest. Exercises promoted sensorimotor integration, functional muscle activation and were task-dependent, reflecting common everyday tasks including: reaching, grasp and release, rotation and object manipulation, as described by a previous motor relearning program (Carr and Shepherd, 2003). Examples of some exercises prescribed are shown in Figure 2. Exercises were rated on a 3-point difficulty scale (1 = performed with ease; 2 = performed with some difficulty; 3 = cannot perform) to ensure appropriate progressive overload and prevent ceiling effects.

### tDCS Protocol

bihemispheric-tDCS was applied for the initial 20 min of rehabilitation, with a 5 s fade-in fade-out to avoid alternate currents causing transient neuronal firing (Paulus et al., 2008). Two 25 cm^2^ electrodes, soaked in a saline solution (0.9% NaCl), were placed over the M1 representation of the extensor carpi radialis (ECR) on each hemisphere, and secured with a rubber strap. The optimal ECR hotspot for each hemisphere was explored and determined with TMS and marked for the entire intervention to ensure consistent electrode placement. In both conditions, the anode was over the M1 contralateral to the paretic limb and the cathode over the M1 contralateral to the non-paretic limb. The stimulation was delivered at 1.5 mA (current density 0.06 mA/cm^2^) through a DC-stimulator (NeuroConn DC stimulator, Ilmenau, Germany). Double-blinding was achieved through a coded device, allowing for real and pseudo stimulation. In the sham-tDCS group, stimulation ceased after 5 s providing a pseudo-stimulation effect. A 10-point (0 = no sensation to 10 = extremely painful) visual analog scale (VAS) was used during the first 3 min of stimulation in week 1 in order to determine the perceived sensation between groups.

### Surface Electromyography

Surface Electromyography (sEMG) was recorded using bipolar Ag-AgCL electrodes. Two electrodes were placed 2 cm apart on the mid belly of the ECR, with a ground strap placed around the wrist as a common reference for electrodes. Cables were fastened with tape to prevent movement artifact. The skin was prepared prior to electrode placement to ensure a clear signal. sEMG signals were amplified (×100–1000), bandpass filtered (high pass at 13 Hz, low pass at 1000 Hz), digitized online at 2 kHz for 500 ms, recorded and analyzed using PowerLab 4/35 (ADInstruments, Bella Vista, NSW, Australia).

### TMS Protocol

TMS was delivered over the cortical representation of the ECR, using a figure-of-eight coil (external diameter 90 mm) attached via a BiStim unit, to 2 Magstim 200^2^ stimulators (Magstim, Dyfed, UK). The coil was positioned tangentially over the M1 at a 45° angle in a posterior-anterior direction. Sites near the estimated center of the ECR were explored, and the largest most consistent motor evoked potential (MEP) amplitude was marked with an “X”. The researcher maintained this mark throughout the intervention to ensure consistency and reliability of coil placement. Measurements included active motor threshold (AMT), MEP amplitudes and cortical silent period (CSP) duration at 150% AMT, 120% AMT, as well as short-interval intracortical inhibition (SICI). AMT was defined as the minimum stimulator intensity which produced an MEP amplitude of >200 μV, in at least 5 out of 10 stimuli. MEP amplitudes and CSP were measured by delivering 10 stimuli at a stimulator intensity equivalent to 150% AMT, to allow for a minimum of 5 consistent MEPs to be obtained. To maintain a constant level of background muscle activity, visual feedback of muscle rmsEMG was displayed on an oscilloscope (HAMEG, Mainhausen, Germany) and participants were asked to maintain a light contraction no greater than 5% ± 2 of maximal rmsEMG. Pre stimulus rmsEMG of the ECR was obtained 100 ms prior to each TMS stimulus and MEPs with pre stimulus rmsEMG that exceeded 5% ± 2 maximal rmsEMG, were discarded and repeated at the appropriate intensity (Sale and Semmler, 2005). SICI was obtained by delivering a conditioning stimulus at 80% AMT then a test stimulus of 120% AMT, separated by a 3 ms inter-stimulus interval (ISI; Zoghi et al., 2003; Garry and Thomson, 2009). Ten test and conditioned stimuli were delivered, with a rest period of 30 s between stimuli sets to avoid muscular fatigue. For the paired-pulse paradigm, both the test and conditioning stimulator intensities were adjusted if any changes in AMT were observed, so that the MEP amplitudes were always equivalent to the true percentage of AMT.

Direct muscle responses (M-waves) were recorded by direct supramaximal electrical stimulation (pulse duration 1 ms) of the radial nerve under resting conditions. A high-voltage constant current stimulator (DS7, Digitimer^®^, Hertfordshire, UK) delivered each electrical pulse. Bipolar electrodes were positioned over the radial nerve on the distal, lateral shaft of the humerus and the stimulation intensity was increased by 5% increments until there was no further increase in sEMG amplitude (M_MAX_). The intensity was increased an additional 20% and the average M_MAX_ obtained from 5 stimuli was delivered and recorded at 0.2 Hz. All TMS and M-wave procedures were performed for both limbs and the order of limb testing was randomized.

### Data and Statistical Analysis

**Figure 3:**
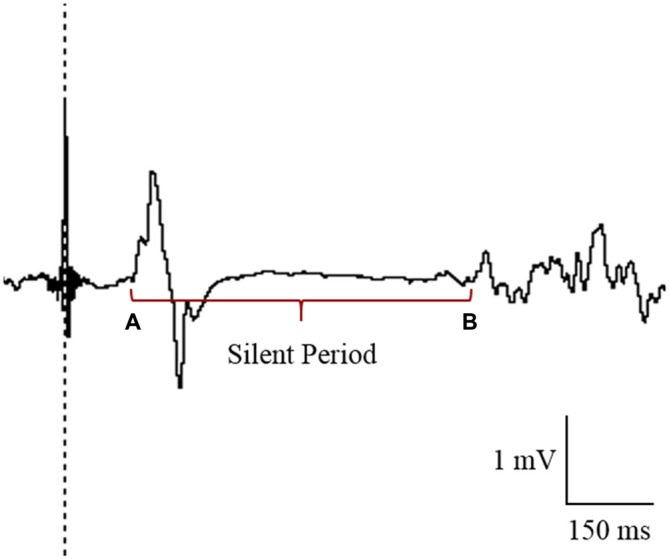
**Cursor placement for the analysis of cortical silent period (CSP) in the contralesional primary motor cortex (M1).** CSP was measured from the onset of the motor evoked potential (MEP; A) to the return of electromyography (EMG; B).

TMS data were analyzed using LabChart 8 Software (ADInstruments, Bella Vista, NSW, Australia). MEPs were quantified by peak-to-peak values (mV) and expressed as a ratio of M_MAX_ for each individual. CSP was recorded as the time (in ms) from MEP amplitude onset to the return of normal EMG activity (Figure 3; Christie and Kamen, 2014). All LabChart files were coded to allow blinding to the tDCS condition during the analysis of CSP. As there was no clear suppression of EMG activity in the paretic limb for the majority of participants, CSPs were only included in analysis for the non-paretic limb. An absence of CSP in the paretic limb has previously been reported in stroke patients (Schnitzler and Benecke, 1994).

SICI was calculated by dividing the raw average conditioned MEP by the raw average test MEP and multiplied by 100.

A laterality index (LI) for interhemispheric asymmetries in corticospinal excitability was calculated on the basis of the mean difference in MEP amplitudes between the 2 hemispheres, following previously published methods in stroke patients (Di Lazzaro et al., 2014). LI ranged from −1 to +1 with a greater distance from 0 representing a larger interhemispheric imbalance. Positive values denote increased excitability of the contralesional M1.

Based on previous data examining motor function and corticospinal excitability in stroke patients (Bolognini et al., 2011), *a priori* power calculations revealed that 14 participants were needed to detect a 20% difference between-groups for these outcomes, assuming a SD of 15–25% with 80% power (two-tailed, *P* < 0.05). Statistical analyses were conducted using Stata statistical software (StataSE version 13). Data was screened with Shapiro-Wilk and due to skewness, all variables except M_MAX_, grip strength, Tardieu scores and LI, were log-transformed before analysis. Independent *t*-tests were used to compare clinical demographics between groups at baseline. For change in spasticity (0–5), a Chi-Square test was used to determine between-group differences in the proportion of participants that had no change, increased or decreased spasticity scores over time. Generalized linear mixed-models were used to assess within-group changes (time) and group-by-time interactions for each of the dependent variables. Within-group changes after 3 and 6 weeks are presented as percentage changes from baseline. The percentage change in the log-transformed measures represent the absolute difference from baseline in log-transformed data multiplied by 100. Between-group differences were calculated by subtracting within-group changes from baseline for the real-tDCS group from within-group changes for the sham-tDCS group. *P* < 0.05 determined statistical significance and data are presented as Mean ± SEM.

## Results

### Participants

Of the 8 participants randomized to both the sham-tDCS and real-tDCS groups, 1 participant withdrew from the sham-tDCS group before the intervention. Intervention compliance was 100% for all participants and no adverse events were reported. There were no between-group differences in any of the clinical demographics (age, *P* = 0.83), (height, *P* = 0.86), (weight, *P* = 0.07), (MMSE, *P* = 0.46) and (years since stroke, *P* = 0.06; Table 1).

**Table 1:** **Mean (± SEM) demographic and clinical characteristics of all participants at baseline**.

Group	Patient ID	Gender	Age	Weight (kg)	Height (cm)	Years since stroke	Lesion site	Handedness	Lesion type	MMSE	MAS score
**Sham-tDCS**
	1	F	46	86	167	3	L:IC	R	H	28	5
	2	F	76	53	149	5	L:MCA	L	H	29	14
	3	F	42	125	165	8	R:MCA	R	I	29	5
	4	F	49	97	172	5	R:ICA	R	I	30	3
	5	F	62	82	157	8	R:MCA	R	I	30	2
	6	M	62	77	176	14	L:MCA	R	H	29	2
	7	M	56	98	183	1	L:PP	R	I	29	13
**Real-tDCS**
	8	F	56	60	165	4	L:MCA	R	H	30	3
	9	M	54	94	179	4	L:MCA	R	I	29	6
	10	F	71	50	154	3	R:ICA	R	I	21	4
	11	F	59	67	165	2	L:MCA	R	I	30	13
	12	M	52	85	182	2	L:SC	R	I	29	2
	13	F	55	53	152	3	L:MCA	R	I	29	5
	14	M	80	85	185	3	L:MCA	L	I	28	9
	15	F	34	60	163	3	L:MCA	R	I	30	5

*M, male; F, female; L, left; R, right; I, ischemic; H, hemorragic; ICA, internal carotid artery; IC, internal capsule; MAS, Motor Assessment Scale; MCA, middle cerebral artery; MMSE, Mini-Mental State Examination; PP, paramedian pontine; SC, striatocapsular*.

### Perceived tDCS Sensation

Mean VAS scores were not different between groups (real-tDCS 2.0 ± 0.5; sham-tDCS, 1.9 ± 0.8; *P* = 0.56).

### Motor Function

#### MAS

**Figure 4:**
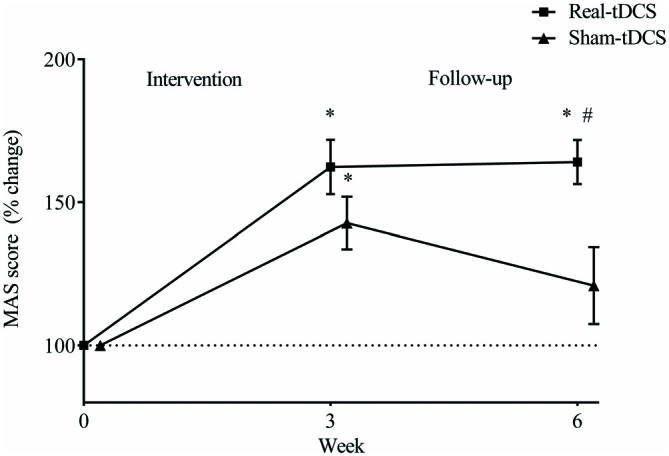
**Mean (± SEM) log Motor Assessment Scale (MAS) scores for the paretic UL.** Results are displayed for post intervention (week 3) and follow-up (week 6) as percentage changes from baseline (week 0). *P < 0.05 within-group change relative to baseline. ^#^*P* < 0.05 between-groups.

Baseline MAS scores were not different between groups (*P* = 0.43). After the 3-week intervention MAS scores improved relative to baseline in both the sham-tDCS (43%) and real-tDCS (62%) groups (*df* = 2, both *P* < 0.001), with no significant group-by-time interaction (Wald Chi-Square = 75.27, *df* = 2, *P* = 0.17; Table 2, Figure 4). After 6-weeks, only the real-tDCS group showed a retention in the MAS improvements (64%, Wald Chi-Square = 13.25, *df* = 2, *P* < 0.001) whereas the gains in the sham-tDCS group began to return to baseline (21%, Wald Chi-Square = 77.21, *df* = 2, *P* = 0.08) which led to a group-by-time interaction (Wald Chi-Square = 75.27, *df* = 2, *P* = 0.002; Table 2, Figure 4).

**Table 2:** **Mean (± SEM) raw values for neurophysiological variables for both limbs in the sham-tDCS and real-tDCS groups for baseline (week 0), immediately post (week 3) and follow-up (week 6)**.

Limb	Group	Time	MAS score	MEP amplitude (% M_MAX_)	CSP (ms)	SICI (% test stimuli)
**Non-paretic**
	**Real**	Baseline	NA	28.0 ± 7.6	124 ± 16.3	64.0 ± 6.3
		Post		20.4 ± 3.4	163 ± 8.7*^#^	55.3 ± 5.3
		FU		26.1 ± 5.5	152 ± 13.5*	50.2 ± 6.9*^#^
	**Sham**	Baseline	NA	34.6 ± 6.9	125 ± 17.7	53.2 ± 10.5
		Post		28.2 ± 6.0	128 ± 15.5	52.8 ± 7.1
		FU		29.7 ± 5.9	132 ± 17.2	52.3 ± 7.0
**Paretic**
	**Real**	Baseline	6 ± 1.3	6.3 ± 1.4	NA	83.9 ± 9.1
		Post	10 ± 1.4*	10.4 ± 2.8*		72.4 ± 4.4
		FU	10 ± 1.5*^#^	9.0 ± 2.1*		71.7 ± 6.7
	**Sham**	Baseline	6 ± 1.9	14.6 ± 2.3	NA	78.6 ± 11.1
		Post	9 ± 2.3*	16.3 ± 2.4		75.4 ± 8.5
		FU	8 ± 2.2	15.5 ± 1.8		72.4 ± 8.6

CSP, cortical silent period; FU, follow-up; MAS, motor assessment scale; MEP, motor evoked potential; M_MAX_, maximal M-wave; NA, not applicable/measured; SICI, short-interval intracortical inhibition. *P < 0.05 compared with baseline (within-group), ^#^P < 0.05 compared with sham (between-group).

#### Grip Strength

Baseline grip strength (N) was not different between groups and did not change over time in either group (Table 2).

#### Tardieu Scale

At baseline, no participants scored higher than “2” on the Tardieu Scale, and the sham-tDCS group had a slightly higher proportion of participants scoring 2 for the wrist (χ^2^ = 6.56, *P* = 0.04) but no group differences at the elbow (χ^2^ = 2.64, *df* = 1, *P* = 0.10). Spasticity did not change over time for either group.

### M_MAX_ and Pre-Stimulus rmsEMG

There were no between-group differences for M_MAX_ and rmsEMG at baseline for either limb. Similarly, there were no within-group changes at 3 or 6-weeks, or group-by-time interactions.

### Corticospinal Excitability

#### AMT

There were no between-group differences at baseline, and no changes were observed over time or between groups after 3 or 6-weeks.

#### MEPs Recorded from the Paretic and Non-Paretic UL (%M_MAX_)

**Figure 5:**
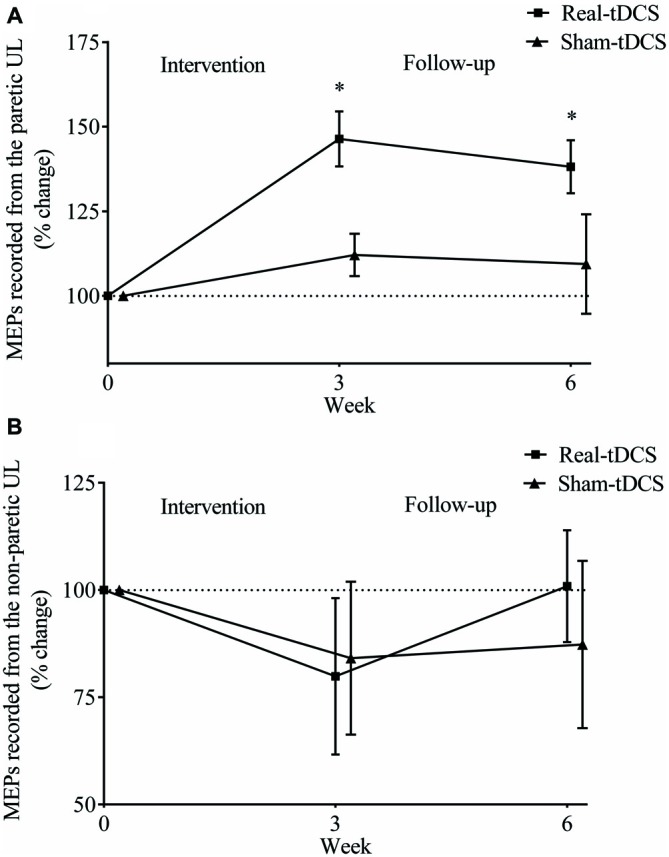
**Mean (± SEM) log motor evoked potential (MEP) amplitudes recorded from the paretic (A) and non-paretic UL (B).** Results are displayed for post intervention (week 3) and follow-up (week 6) as percentage changes from baseline (week 0). *P < 0.05 within-group change relative to baseline.

For the paretic UL, baseline MEP amplitudes were greater in the sham-tDCS group (*P* = 0.01), therefore analysis was adjusted for baseline values and results remained unchanged. After the 3-week intervention, no group-by-time interaction was observed (Wald Chi-Square = 25.65, *df* = 2, *P* = 0.12), but MEPs were facilitated for the real-tDCS group relative to baseline (46%, Wald Chi-Square = 37.49, *df* = 2, *P* < 0.001), with no change for the sham-tDCS (12%, Wald Chi-Square = 0.86, *df* = 2, *P* = 0.36). After 6-weeks, the real-tDCS group maintained larger MEP amplitudes relative to baseline (38%, Wald Chi-Square = 37.49, *df* = 2, *P* < 0.001) with no change in the sham-tDCS group (9%, Wald Chi-Square = 0.86, *df* = 2, *P* = 0.57), but there was no group-by-time interaction (Wald Chi-Square = 25.65, *df* = 2, *P* = 0.09; Table 2, Figure 5A).

For the non-paretic UL, there were no between-group baseline differences (*P* = 0.26) or within-group changes over time or group-by-time interactions (Table 2, Figure 5B).

#### LI

**Figure 6:**
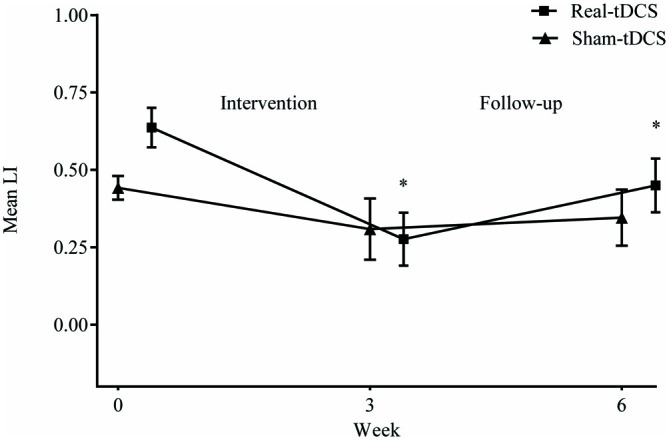
**Mean (±SEM) raw values for laterality index (LI) at baseline (week 0), post intervention (week 3) and follow-up (week 6).** *P < 0.05 within-group relative to baseline.

Baseline LI for both groups combined, shifted towards greater excitability of the ipsilesional M1 (LI = 0.5 ± 0.1) but there were no between-group differences at baseline (*P* = 0.94). Following the 3-week intervention, there was a shift in LI for real-tDCS (from 0.6 to 0.3, Wald Chi-Square = 14.80, *df* = 2, *P* < 0.001), with a trend for a shift in the sham (from 0.4 to 0.3, Wald Chi-Square = 4.45, *df* = 2, *P* = 0.06) but the group-by-time interaction was not significant (Wald Chi-Square = 19.21, *df* = 2, *P* = 0.40). After 6-weeks, the shift in LI for the real-tDCS was maintained relative to baseline (Wald Chi-Square = 14.80, *df* = 2, *P* = 0.03), but there remained no group-by-time interaction (Wald Chi-Square = 19.21, *df* = 2, *P* = 0.97; Figure 6).

### Intracortical Inhibition

#### CSP Recorded from the Non-Paretic UL

**Figure 7:**
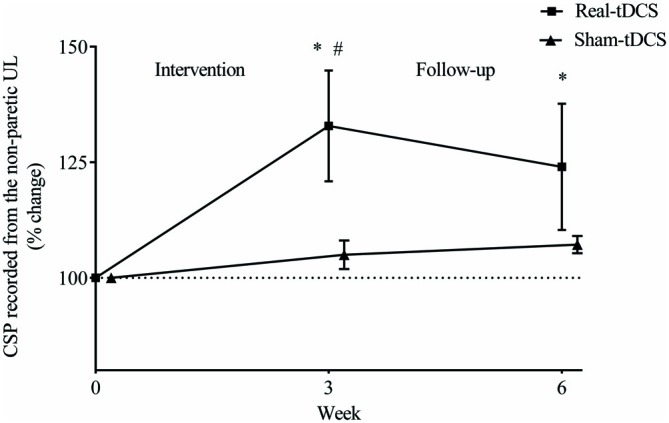
**Mean (± SEM) log CSP recorded from the non-paretic UL.** Results are displayed for post intervention (week 3) and follow-up (week 6) as percentage changes from baseline (week 0). *P < 0.05 within-group change relative to baseline. ^#^*P* < 0.05 between-groups.

There were no baseline differences in the CSP between groups (*P* = 0.49). At 3-weeks, there was a 33% increase in CSP relative to baseline (Wald Chi-Square = 8.16, *df* = 2, *P* = 0.01) in the real-tDCS group with no marked change in the sham-tDCS group (5%, Wald Chi-Square = 2.12, *df* = 2, *P* = 0.32), which led to a group-by-time interaction (Wald Chi-Square = 14.76, *df* = 2, *P* = 0.04; Table 2, Figure 7). After 6-weeks, the increase in the real-tDCS group was maintained relative to baseline (24%, Wald Chi-Square = 8.16, *df* = 2, *P* = 0.04) with no change in the sham (7%, Wald Chi-Square = 2.12, *df* = 2, *P* = 0.16) but no group-by-time interaction (Wald Chi-Square = 14.76, *df* = 2, *P* = 0.22; Table 2, Figure 7).

#### SICI Recorded from the Paretic and Non-Paretic UL

**Figure 8:**
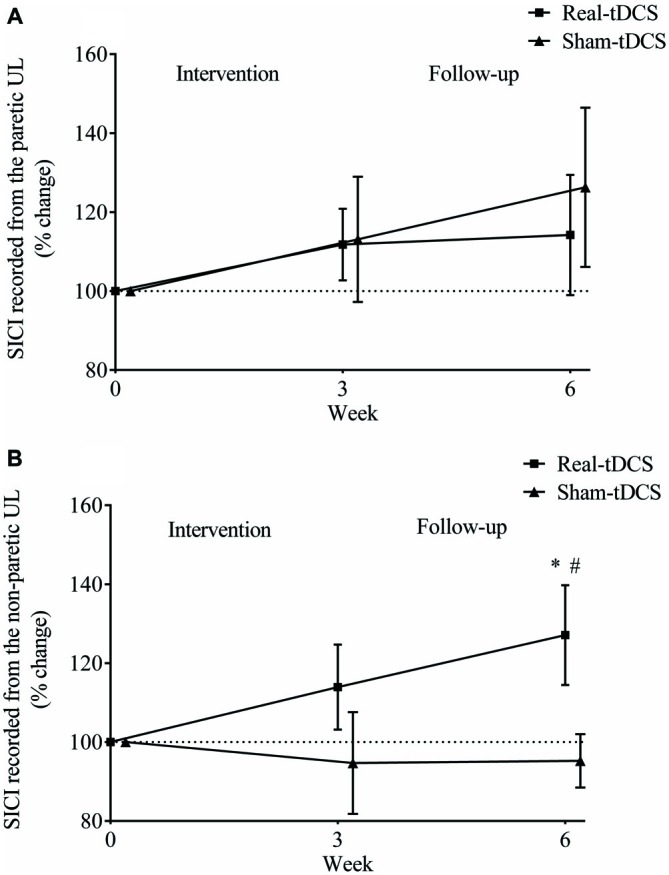
**Mean (± SEM) log short-interval intracortical inhibition (SICI) recorded from the paretic (A) and non-paretic UL (B).** Results are displayed for post intervention (week 3) and follow-up (week 6) as percentage changes from baseline (week 0). *P < 0.05 within-group change relative to baseline. ^#^*P* < 0.05 between-groups.

There were no between-group differences at baseline for SICI recorded from the paretic (*P* = 0.23) or non-paretic ULs (*P* = 0.82). In addition, there was no within-group change or group-by-time interactions for SICI recorded from the paretic UL after 3 or 6-weeks (Table 2, Figure 8A).

Similarly, there were no within-group changes (sham-tDCS, Wald Chi-Square = 0.27, *df* = 2, *P* = 0.64; real-tDCS, Wald Chi-Square = 6.37, *df* = 2, *P* = 0.20) or group-by-time interactions (Wald Chi-Square = 7.08, *df* = 2, *P* = 0.22) for SICI recorded from the non-paretic UL after 3-weeks. However after 6-weeks, there was a 27% increase in SICI in the real-tDCS compared with sham-tDCS (group-by-time interaction, Wald Chi-Square = 7.08, *df* = 2, *P* = 0.04; Table 2, Figure 8B).

## Discussion

The main findings were that there was no marked effect of real bihemispheric-tDCS on the immediate improvements in motor function, but it did promote greater retention of MAS gains. Furthermore, real-tDCS increased corticospinal excitability within the ipsilesional M1 and intracortical inhibition within the contralesional M1, which improved hemispheric balance. These findings provide evidence for the efficacy of bihemispheric-tDCS combined with UL rehabilitation to promote corticospinal plasticity within both the ipsi- and contralesional M1, which appears to be an important process for retaining gains in motor function following stroke.

### Motor Function

Immediately following the intervention, UL rehabilitation elicited an improvement in the MAS, independent of the tDCS group. However 3-weeks following the intervention, real-tDCS produced greater retention of motor function compared with sham-tDCS. While several previous short-term studies (single or 5 consecutive sessions) have also reported additive benefits on motor function immediately following bihemispheric-tDCS and physical therapy compared to physical therapy alone (Lindenberg et al., 2010; Lefebvre et al., 2012, 2014, 2015), the longer rehabilitation period in this study likely contributed to the greater overall practice effect on motor function, independent of bihemispheric-tDCS.

A novel finding in the current study was that there was a preferential effect of bihemispheric-tDCS in the maintenance of motor function, which is in line with previous studies following single and repeated bihemispheric-tDCS sessions (Lindenberg et al., 2010; Bolognini et al., 2011; Lefebvre et al., 2014, 2015). However contrary to these previous studies, the greater retention observed following bihemispheric-tDCS in this study occurred irrespective of similar improvements between-groups immediately following rehabilitation. These findings indicate that the additive effect of bihemispheric-tDCS to rehabilitation may be markedly important for maintaining newly regained motor skills. For example, Bolognini et al. (2011) prescribed a similar number of bihemispheric-tDCS sessions (10 sessions across 2-weeks) and demonstrated that bihemispheric-tDCS with CIMT modulated inhibitory networks within inter-hemispheric pathways (Bolognini et al., 2011), which appears critical for motor learning after stroke. As retention of neurophysiological adaptations were not assessed in this previous study, our findings are the first to demonstrate the preferential effect of bihemispheric-tDCS on maintaining functional improvement, through modulating plasticity within the ipsi- and contralesional corticospinal pathways. Given the evidence for LTP-like plasticity following repeated sessions of tDCS (Monte-Silva et al., 2013), it can be speculated that the addition of bihemispheric-tDCS during recurrent rehabilitation sessions may have improved the retention of motor function through LTP-like mechanisms.

It is interesting to note that although the rehabilitation exercises were reflective of the muscle synergies utilized in the MAS, the exercises were not identical, suggesting some transfer of motor function to untrained tasks. These findings have been supported in previous studies, whereby bihemispheric-tDCS improved performance on range of common daily activities not specifically trained in the intervention (Waters-Metenier et al., 2014; Lefebvre et al., 2015). Taken together, the lasting improvements in motor function following bihemispheric-tDCS and UL exercise, has important clinical implications for improving the long term efficacy of current treatments in stroke rehabilitation.

As expected, we observed no changes in grip strength or spasticity concurrent with improvements in the MAS. In this study, the improvements on the MAS independent of spasticity supports previous findings (Ada et al., 2006), indicating the mechanisms modulating motor function and spasticity may be mutually exclusive, and were not likely affected by bihemispheric-tDCS in this study.

### Corticospinal Excitability and Inhibition

Our findings demonstrate that an increase in inhibition (CSP and SICI) within the contralesional M1 following bihemispheric-tDCS and rehabilitation may be an important mechanism contributing to the lasting gains in motor function. Although only speculative, increasing inhibition in the contralesional M1 may have provided an opportunity for greater activation of the ipsilesional M1 during the rehabilitation tasks, and thus contributed to an overall increase in corticospinal excitability of the paretic UL.

Although we observed no between-group interactions for MEP amplitudes from the paretic UL, it is worth noting that the improvement in the real-tDCS group following the intervention was nearly four-fold greater than the gain in the sham-tDCS group. These findings are consistent with many previous TMS studies (Bolognini et al., 2011; Di Lazzaro et al., 2014), as well as fMRI studies reporting greater activation in the ipsilesional hemisphere following tDCS (Lindenberg et al., 2010; Stagg et al., 2012). The increase in corticospinal excitability within the ipsilesional M1 outlasted the stimulation period up to 3-weeks, which reflects improved synaptic efficacy along the corticospinal pathway corresponding to the paretic UL. Certainty, these mechanisms have been described in both healthy adults following both motor learning (Ziemann, 2004) and tDCS (Liebetanz et al., 2002; Nitsche et al., 2003). Therefore, it appears that the combination of bihemispheric-tDCS with rehabilitation improved the net corticospinal excitability in the ipsilesional M1, leading to lasting improvements in motor function.

The measurement of both SICI and CSP represent local GABA_A_ and GABA_B_ interneuron activity respectively (Byrnes et al., 2001). Contrary to our hypothesis, we observed no change in SICI recorded from the paretic UL. This finding is also in contrast to previous studies in stroke utilizing a-tDCS, which have reported reductions in SICI corresponding with improvements in motor function (Hummel et al., 2005; Edwards et al., 2009; Honaga et al., 2013). Moreover, MRI studies have demonstrated inverse correlations between functional improvement and individual reductions in GABA levels within the M1 (Kim et al., 2014; Blicher et al., 2015). One possible explanation as to why our study observed no significant change in SICI within the ipsilesional M1, may be the current flow during the application of bihemispheric-tDCS compared with other electrode montages. During bihemispheric-tDCS (M1-M1 arrangement) the current flow differs from a-tDCS (M1-Supraorbital arrangement; Faria et al., 2011), and may generate electrical activity onto adjacent, interconnected regions outside the M1 (Schlaug et al., 2008). Evidence from fMRI has demonstrated ipsilateral and interhemispheric connectivity between the ipsilesional M1 and the supplementary motor area (SMA; Park et al., 2011; Rosso et al., 2013; Grefkes and Fink, 2014), primary motor cortex (PMC; Seitz et al., 1998; Sharma et al., 2009) cerebellum (Rosso et al., 2013) and thalamus (Park et al., 2011; Young et al., 2014). Moreover, the relatively large and non-focal nature of the tDCS electrodes may have additionally targeted activity of remote neuronal tissue (Nitsche et al., 2007; DaSilva et al., 2011). Therefore, it is possible that improved synaptic efficacy from surrounding motor areas may have additionally contributed to the net excitability of the ipsilesional corticospinal pathway in this study.

Consistent with a previous study in acute stroke (Di Lazzaro et al., 2014), the addition of bihemispheric-tDCS resulted in more balanced hemispheric LI, driven primarily by increased corticospinal excitability of the ipsilesional M1. As no significant suppression of MEP amplitude was observed in the contralesional M1, increased ipsilesional excitability may have been mediated through changes in local inhibition within the contralesional M1 (Bolognini et al., 2011; Di Lazzaro et al., 2014). Previous bihemispheric-tDCS studies in chronic stroke have attributed functional recovery to a reduction in IHI from the contra- to the ipsilesional M1 (Bolognini et al., 2011; Di Lazzaro et al., 2014). However there is limited evidence as to the contribution of intracortical inhibition within the contralesional M1 on retaining motor function. One previous study demonstrated no change in CSP following a single session of both a-tDCS and c-tDCS (Suzuki et al., 2012). In contrast, we demonstrated that following 9 sessions, CSP recorded from the non-paretic UL increased and was sustained at follow-up in the real-tDCS group. The CSP increased in the absence of any significant suppression of MEP amplitudes from the non-paretic UL which supports previous work in healthy individuals (Wilson et al., 1993), suggesting these parameters are not directly correlated and may be modulated through different neuronal circuits. Certainly, bihemispheric-tDCS may have a pronounced influence on the activity of intracortical inhibitory neurons (Lang et al., 2011), which may exert a neuromodulatory effect on the ipsilesional M1, through transcallosal pathways (Lang et al., 2004).

Interestingly, SICI recorded from the non-paretic UL was only significantly increased at the follow-up, which suggests an offline neuromodulatory effect of bihemispheric-tDCS that occurred after the cessation of the intervention. In healthy individuals, offline tDCS effects have been suggested to consolidate synaptic plasticity and LTP-like processes (Galea and Celnik, 2009; Janine Reis et al., 2009; Fritsch et al., 2010), which are important for improving and retaining motor learning. Therefore, it is plausible that bihemispheric-tDCS applied during UL rehabilitation, may have augmented the excitatory response in the ipsilesional M1 from the rehabilitation itself, through up-regulating GABA-ergic inhibition within the contralesional M1.

Following stroke, disinhibition of the contralesional M1 is often indexed by a down-regulation of GABAergic inhibitory activity (Que et al., 1999; Reinecke et al., 1999), but it remains unclear as to how this influences motor function. Longitudinal studies have demonstrated that the restoration of SICI to normal levels within the contralesional M1 is associated with improved functional recovery, whereas individuals with greater disability display abnormally low levels of SICI within the contralesional M1 (Liepert et al., 2000a; Manganotti et al., 2002, 2008). Therefore, the increase in intracortical inhibition observed at the follow-up period may be a key mediator for the lasting improvements in motor function following bihemispheric-tDCS and rehabilitation. Furthermore, evidence for compensatory mechanisms within the contralesional M1 highlight the influence of the ipsilateral corticospinal pathway on motor function of the paretic limb (Farias da Guarda et al., 2010). Disinhibition within the contralesional M1 may interfere with motor function of the paretic limb, through an inability to supress antagonist muscle activation and control muscle synergies (Schwerin et al., 2008; Bradnam et al., 2010). In healthy adults, c-tDCS improved selective muscle activation through inhibition of the antagonist muscle in the proximal UL (McCambridge et al., 2011; Uehara et al., 2015). In stroke, these processes are dependent on the degree of spasticity (Bradnam et al., 2012) and corticospinal pathway damage (Ward et al., 2007; Bradnam et al., 2012), which may contribute to variability in the responsiveness to bihemispheric-tDCS. Although these previous studies assessed proximal muscles, it can be speculated that the increase in contralesional M1 inhibition observed in this study, may have contributed to improved motor control of the paretic UL through the ipsilateral uncrossed corticospinal pathway.

### Limitations

As this study was an exploratory pilot study, we included a heterogeneous sample of stroke patients in order to maximize the generalizability of tDCS use across a stroke affected population. However, results should be viewed with caution as variation between patients may influence the responsiveness to bihemispheric-tDCS and contribute to inter-participant variability. Future research should aim to perform subgroup analyses amongst different strong etiologies, in order to assess the efficacy of tDCS across a diverse cohort of stroke patients. Nevertheless, the results from this study demonstrate significant retention of motor function following the combination of bihemispheric-tDCS and UL rehabilitation, suggesting this mode of rehabilitation may be feasible across chronic stroke patients. These findings warrant larger trials to investigate individualizing tDCS treatment in different subgroups of stroke. Considering the heterogeneous nature of stroke and variability of tDCS responsiveness, multicenter clinical trials with larger sample sizes and longer follow-up periods are needed to determine the clinical efficacy of bihemispheric-tDCS in stroke rehabilitation. Finally, although we observed a shift in the LI towards hemispheric balance, we were unable to quantify IHI, which has been shown to be a key pathway modulated during bihemispheric-tDCS (Bolognini et al., 2011; Di Lazzaro et al., 2014).

## Conclusion

Our findings indicate the potential for bihemispheric-tDCS to improve retention of UL motor function in chronic stroke patients. Increased inhibition within the contralesional M1 may have subsequently amplified the excitatory effects of the anode and improved retention of motor function in this cohort of stroke survivors. Given the varied stroke etiologies, our findings warrant larger clinical trials to identify the variables influencing individual responsiveness to tDCS, tailoring the stimulation parameters based on these cofounders. Moreover, the optimal intensity of concurrent rehabilitation provided needs to be identified in order to improve efficacy and generalizability across a broad range of stroke-affected individuals.

## Author Contributions

All experiments were conducted in the neurophysiology and exercise laboratory at Deakin University, Burwood, Victoria, Australia. AMG was involved in the conception and design of the experiment as well as collecting, analyzing and interpreting the data. AMG additionally led the drafting and critical revision of the article. DJK was involved in the conception and design of the experiment, interpretation of data and critical revision of the article. W-PT was involved in the conception and design of the experiment, analyses and interpretation of the data and critical revision of the article. RMD was involved in the conception and design of the experiment, analyses and interpretation of the data and critical revision of the article. PM was involved in the conception and design of the experiment as well as critical revision of the article.

## Conflict of Interest Statement

The authors declare that the research was conducted in the absence of any commercial or financial relationships that could be construed as a potential conflict of interest.
